# A multi-distance laser-induced breakdown spectroscopy data classification method based on deep convolutional neural network and spectral sample weight optimization

**DOI:** 10.1038/s41598-025-24644-x

**Published:** 2025-11-19

**Authors:** Xuchen Zhang, Luning Li, Zhicheng Cui, Weiming Xu, Xuesen Xu, Rong Shu, Xiangfeng Liu, Jianyu Wang

**Affiliations:** 1 Key Laboratory of Space Active Opto-electronics Technology, Shanghai Institute of Technical Physics, Chinese Academy of Sciences, Shanghai, 200083 China; 2 Hangzhou Institute for Advanced Study, University of Chinese Academy of Sciences, Hangzhou, 310024 China; 3 University of Chinese Academy of Sciences, Beijing, 100049 China; 4 Innovation Academy for Microsatellites, Chinese Academy of Sciences, Shanghai, 201304 China

**Keywords:** Laser-induced breakdown spectroscopy, Convolutional neural network, Multi-distance spectra, Spectral sample weight optimization, MarSCoDe, Engineering, Mathematics and computing, Optics and photonics

## Abstract

Laser-induced breakdown spectroscopy (LIBS) is a stand-off chemical analysis technique. In scenarios where the LIBS detection distance varies (e.g. Mars exploration), the distance effect poses a significant challenge to data analysis. In our prior work, a deep convolutional neural network (CNN) model was developed to directly process LIBS multi-distance spectra, achieving high classification accuracy even without performing conventional “distance correction”. The present study proposes a spectral sample weight optimization strategy to further improve the CNN model training process. Unlike the default equal-weight scheme, the new strategy tailors a specific weight value for every training spectral sample. On an eight-distance LIBS dataset acquired by the MarSCoDe duplicate instrument, the CNN model with the new weighting strategy can achieve a maximum testing accuracy of 92.06%, representing an improvement of 8.45 percentage points over our original CNN model. Besides accuracy, three other supplementary metrics also demonstrate the superiority of the new strategy: the precision, recall and F1-score can be averagely increased by 6.4, 7.0 and 8.2 percentage points, respectively. Moreover, the training time per epoch of the weight optimization strategy is almost identical to that of the original equal-weight scheme. These results indicate that the proposed methodology has great application potential in planetary exploration, and other LIBS-adopted scenarios involving varying detection distances.

## Introduction

Laser-induced breakdown spectroscopy (LIBS), an advanced atomic emission spectrochemical technique, exhibits exceptional capability in stand-off and in-situ detection of solid, liquid, gaseous, and colloidal specimens^1^. The LIBS technique utilizes high-energy laser pulses to ionize the atoms in the substance and generate plasma emission. In a LIBS spectrum, the wavelengths of the characteristic spectral lines contain fingerprint signatures for element identification, while the intensities of the spectral lines provide information on element concentrations^2,3^.

**Fig. 1:**
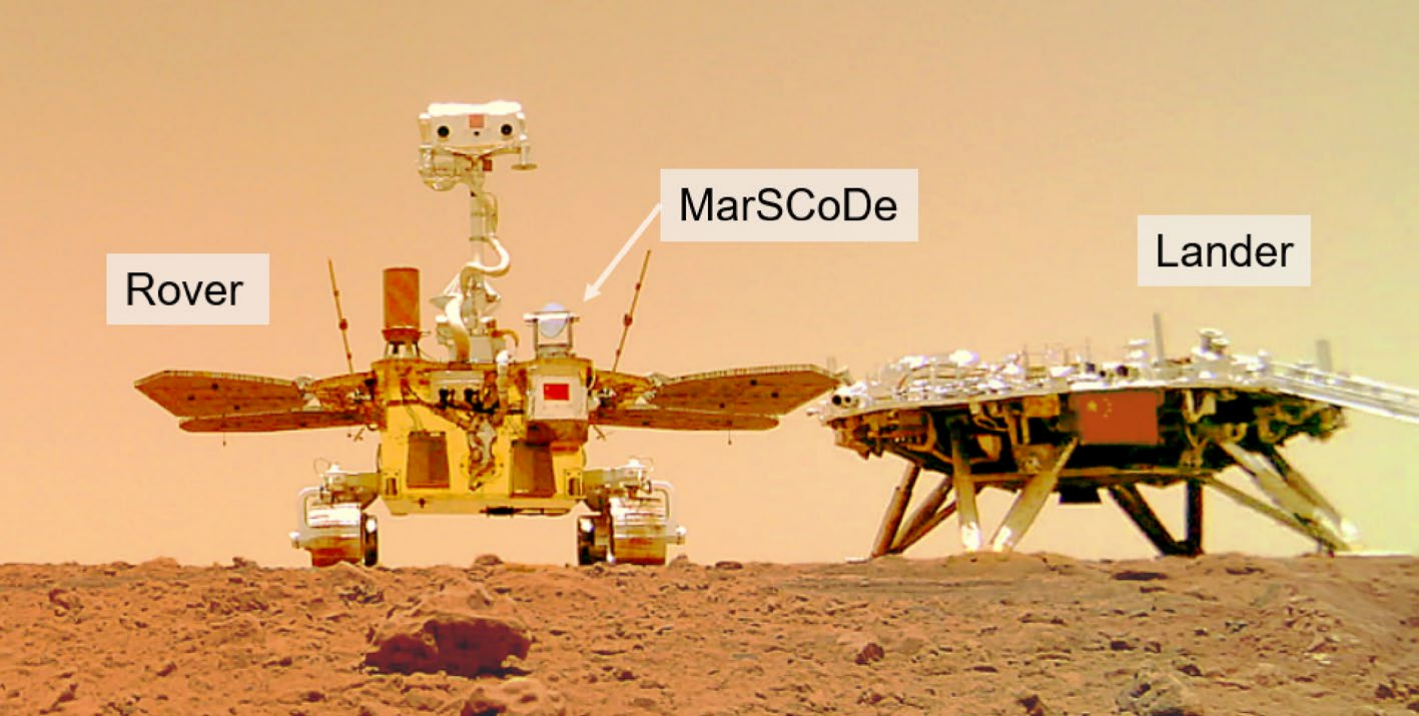
Diagram of China’s Tianwen-1 Mars Lander, Zhurong Rover, and the MarSCoDe instrument on the rover fore deck. The original picture is available on the Internet, provided by China’s Lunar Exploration and Space Engineering Center.

LIBS has been widely applied in environmental monitoring^4,5^, industrial control^6^, nuclear engineering^7^, biomedical research^8,9^, planetary exploration, etc. Particularly in planetary exploration, LIBS has become the key technique for analyzing planetary surface materials due to its non-contact and remote detection capabilities^10–12^. LIBS instruments have been equipped on three Mars rovers, namely NASA’s Curiosity rover (ChemCam)^13,14^, Perseverance rover (SuperCam)^15,16^, and China’s Zhurong rover (MarSCoDe) in Tianwen-1 Mission^17,18^. Figure 1 illustrates the Tianwen-1 Mars lander, Zhurong rover, and the rover-mounted MarSCoDe instrument. By leveraging chemometrics methods, the MarSCoDe LIBS system is able to realize identification and classification of geochemical targets, as well as quantitative analysis of elemental compositions in Martian surface targets.

The LIBS detection distance is usually defined as the distance between the laser and the target sample^19^. In laboratory LIBS measurements, the detection distance can be precisely controlled and fixed. However, in practical Mars exploration scenarios, the detection distance naturally varies from time to time. The distance variations may alter multiple parameters, including laser spot size and energy distribution, geometric configuration of plasma generation zones, absorption/scattering effects of environmental media on excitation and emission radiation, as well as plasma temperature and electron density^20–22^. These collective variations induce the LIBS distance effect, i.e. even when the same target sample is detected by a fixed LIBS system, considerable spectral profile discrepancies can appear if the distance changes, such as intensity variations in characteristic spectral lines, continuum background baseline shifts, and altered elemental peak I’ntensity ratios^23,24^.

Such distance-dependent spectral deviations can weaken the performance of LIBS chemometrics models, as most algorithms require abundant LIBS data collected under identical experimental conditions as training set to ensure good learning effectiveness and robust generalizability. However, planetary field detection can hardly provide sufficient LIBS spectra collected at a fixed distance^25^.

A viable approach to enhancing the LIBS chemometrics performance is to train the model with multi-distance spectral data. In order to effectively utilize such LIBS multi-distance mixed spectra, the ChemCam team has designed several schemes to perform distance correction. These distance correction schemes, either based on spectral response-based correction functions^26^, or distance calibration curve-based protocol^27^, can partially mitigate the profile discrepancy between the various-distance spectra. Nevertheless, the distance correction methodology typically requires designing a specific correction model for each element, and no universal framework has been successfully established. This element-specific property further renders the distance correction a kind of laborious and time-consuming methodology.

Another kind of methodology does not rely on distance correction. In our previous work, we developed a deep convolutional neural network (CNN) model capable of directly analyzing LIBS multi-distance mixed spectra^25^. When tested on a multi-distance spectra dataset without any distance correction, the deep CNN model achieved higher classification accuracy than four other regular chemometrics models, namely back-propagation neural network, support vector machine, linear discriminant analysis and logistic regression.

While the deep CNN methodology demonstrates high accuracy and efficiency, its training process has potential limitations and there is still room for improvement. Specifically, in the training process of that CNN model, a uniform sample weighting strategy was employed by default, i.e. equal weight was assigned to each spectral sample in the training set. The spectral feature disparities induced by varying distances were not sufficiently considered.

In this study, we propose a new sample weight strategy for training the deep CNN model. Each spectral sample in the training set is assigned a tailored weight, which is calculated based on the detection distance corresponding to the sample. On the same LIBS multi-distance spectra dataset, the equal-weight scheme and the proposed optimized-weight scheme are compared in terms of classification performance.

In the following text, in “Experiment” section briefly describes the LIBS experiment and the multi-distance spectra dataset. In “Classification method” section presents the architecture of the implemented deep CNN model, with particular emphasis on the novel spectral sample weight optimization strategy. In “Results and discussion” section offers the exhibition and explanation of the results and provides a further discussion. A conclusion can be found in “Conclusion” section.

## Experiment

In “Experiment” section, the information about the LIBS instrument, target samples, and datasets is provided.

### LIBS instrument

The LIBS instrument employed in this experiment is a laboratory duplicate model of the MarSCoDe payload. The technical specifications of the MarSCoDe duplicate model are highly similar to those of the genuine payload onboard the Zhurong rover (as described in^17^). For the MarSCoDe instrument, the LIBS excitation source is a Nd:YAG laser with a wavelength of 1064 nm, a pulse energy of 9 mJ, a pulse width of 4 ns and a pulse repetition rate of 1–3 Hz, and the detection distance ranges from 1.6 to 7 m. The LIBS spectrometer system contains three spectral channels, covering three wavelength ranges, namely 240–340 nm, 340–540 nm and 540–850 nm. The spectrometer slit width is 14 μm. For the gratings of the three spectral channels, the groove densities are 1500 mm^−1^, 768 mm^−1^ and 500 mm^−1^, respectively, and the blaze wavelengths are approximately 250 nm, 430 nm and 650 nm, respectively. Each channel contains 1800 pixels and hence each raw LIBS spectrum consists of 5400 pixel data points.

### Target samples and class definition

There are 37 target samples used in this study, selected from the 39 samples adopted in our prior work^25^ (Sample No. 38 and Sample No. 39 therein are excluded in this study). All the target samples are certified Chinese national reference materials (GBW series), with relevant information displayed in Table 1. Initially existing as homogeneous powders, these materials have been processed into tablets through a standardized compression molding process.

**Table 1:** The 37 national standard reference materials employed in the experiment, including rocks, soils, sediments and ores.

No.	Material	Reference ID	No.	Material	Reference ID
1	Clay	GBW03101a	20	Saline-alkali soil type-I	GBW07447
2	Soft clay	GBW03115	21	Saline-alkali soil type-II	GBW07449
3	Carbonate rock	GBW07127	22	Sierozem	GBW07450
4	Kaolin	GBW03121a	23	Beach sediment	GBW07453
5	Basalt	GBW07105	24	Granite	GBW07103
6	Pegmatite	GBW07125	25	Shale type-II	GBW07107
7	Dolomite	GBW07217a	26	Nickel ore	GBW07146
8	Andesite	GBW07104	27	Polymetallic lean ore	GBW07162
9	Granitic gneiss	GBW07121	28	Copper rich ore	GBW07164
10	Siliceous sandstone	GBW03112	29	Lead‐zinc rich ore	GBW07165
11	Shale type-I	GBW03104	30	Lead ore type-I	GBW07235
12	Quartz sandstone	GBW07106	31	Lead ore type-II	GBW07236
13	Argillaceous limestone	GBW07108	32	Molybdenum ore	GBW07239
14	Polymetallic ore	GBW07163	33	Stream sediment type-III	GBW07305a
15	Stream sediment type-I	GBW07309	34	Stream sediment type-IV	GBW07307a
16	Stream sediment type-II	GBW07377	35	Stream sediment type-V	GBW07308a
17	Floodplain sediment	GBW07390	36	Stream sediment type-VI	GBW07310
18	Yellow–red soil	GBW07405	37	Stream sediment type-VII	GBW07311
19	Latosol	GBW07407			

The target sample classes are defined using the following method. The primary strategy utilizes the results from K-Means clustering and principal component analysis (PCA). Each of the 37 target samples is characterized by a 1 × 8 vector, based on the concentration values of eight chemical components (SiO_2_, Al_2_O_3_, MgO, Na_2_O, K_2_O, TiO_2_, FeO_T_, and CaO). Then K-Means is used to perform cluster analysis on these vectors. Finally, PCA is employed to visualize the clustering results. In the two-dimensional PCA plot, each dot represents a target sample, and those dots naturally forming a clear cluster are regarded as one class. However, some dots cannot be easily assigned to a single cluster because their intra-cluster distance is too large and/or their inter-cluster distance is too small. Hence we have also used a secondary strategy based on specific geochemical characteristics of the target samples (i.e. the concentration of specific element or component), as exemplified below.

The class definition scheme is shown in Table 2. The 37 geochemical samples are categorized into six classes, namely Carbonate Mineral, Regular Rock, Clay, Regular Soil, Metal Ore, and High-silica Rock. The first four classes are defined based on the primary strategy, while the last two classes are defined based on the secondary strategy. For the samples belonging to Metal Ore, each sample must contain at least 0.1 wt% specific metallic elements. For example, the concentration of Mo in Sample No. 32 (Molybdenum ore) is approximately 0.11 wt%. As to the samples belonging to High-silica Rock, each sample must contain at least 70 wt% SiO_2_ component. For example, the concentration of SiO_2_ in Sample No. 24 (Granite) is approximately 72.83 wt%.

**Table 2:** The class definition scheme for the 37 geochemical samples.

Class no. and name	Sample no.
(1) Carbonate mineral	3, 7, 13
(2) Regular rock	5, 8, 9, 11, 16, 23, 25, 27
(3) Clay	1, 2, 4, 18, 19
(4) Regular soil	15, 17, 20, 21, 22, 26, 33, 34
(5) Metal ore	14, 28, 29, 30, 31, 32
(6) High-silica rock	6, 10, 12, 24, 35, 36, 37

### LIBS datasets

As mentioned above, the LIBS spectra were acquired using the MarSCoDe duplicate model. The measurements were conducted under standard laboratory conditions, with both the LIBS instrument and the target samples placed in an ordinary atmospheric environment. During the LIBS measurements, the gate delay and the gate width were set to 0 μs and 1000 μs (1 ms), respectively.

The multi-distance spectra dataset employed in this study comprises eight distinct distances, roughly including three types: short, medium, and long ranges. The short-range distances are 2.0 m (Distance 1), 2.3 m (Distance 2), and 2.5 m (Distance 3); the medium-range distances are 3.0 m (Distance 4), 3.5 m (Distance 5), and 4.0 m (Distance 6); while the long-range distances are 4.5 m (Distance 7) and 5.0 m (Distance 8). For each distance, 60 LIBS spectra were collected from each target sample. Therefore, 2220 spectra were acquired from all the 37 target samples for one distance. The spectra dataset collected at Distance 1 is defined as “d1”, the dataset collected at Distance 2 is defined as “d2”, …, and the dataset collected at Distance 8 is defined as “d8”. The whole eight-distance mixed spectra dataset encompasses 17,760 spectra.

Each raw LIBS spectrum has undergone a series of preprocessing procedures, including dark background subtraction, wavelength calibration, ineffective pixel masking, spectrometer channel splicing, and background baseline removal. A noteworthy issue concerns the number of data points per spectrum. As mentioned in “LIBS instrument” section, each raw spectrum consists of 5400 pixel data points. However, after the wavelength calibration (establishment of the pixel-to-wavelength relationship), in each channel there are some pixels corresponding to “ineffective” wavelength values (i.e. beyond the three wavelength ranges mentioned above). Therefore, those pixels are regarded as ineffective pixels and the corresponding data points are removed. After such an ineffective pixel masking, each preprocessed spectrum actually includes 4514 pixel data points. Consequently, each spectral sample is represented by a 4514 × 1 vector in the subsequent computation.

**Fig. 2:**
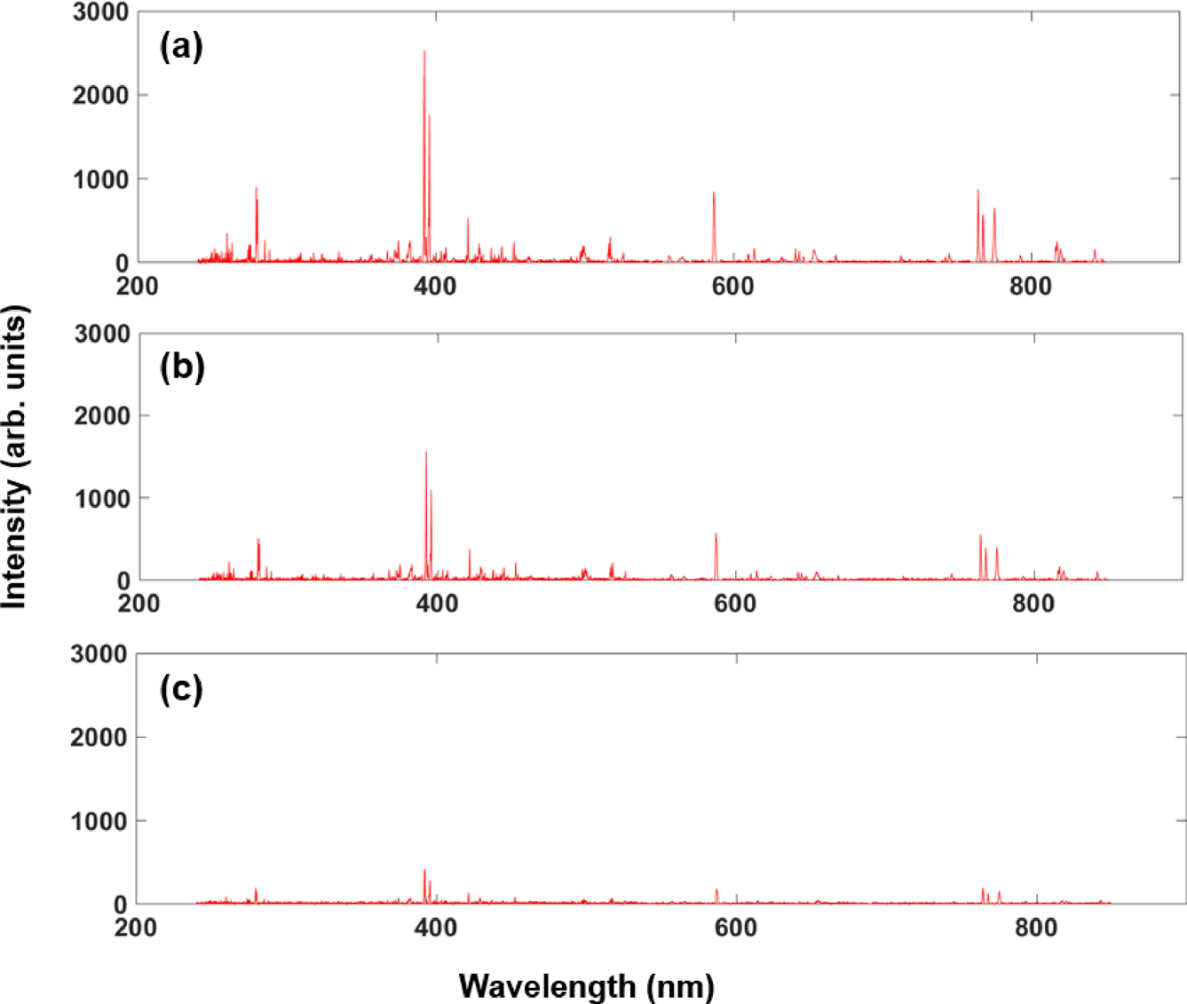
Typical LIBS preprocessed spectra of Sample No.5 (Basalt) collected at three different distances: (**a**) 2.3 m, (**b**) 3.0 m, (**c**) 4.5 m. For each detection distance, the illustrated spectrum is randomly picked out from the 60 collected spectra.

In Fig. 2, three typical LIBS spectra after the preprocessing procedures are exhibited. Figure 2a shows a preprocessed spectrum of Sample No.5 (Basalt) collected at 2.3 m (short-range), while Fig. 2b and c correspond to the spectra of the same target sample collected at 3.0 m (medium-range) and 4.5 m (long-range), respectively. It can be found that the alteration of the detection distance does not change the wavelength positions of the characteristic emission lines, indicating the stable response performance of the LIBS instrument. On the other hand, as the distance increases, the line intensities present a trend of attenuation, with the signal-to-noise ratio (SNR) values progressively decreasing.

## Classification method

As stated before, this study further enhances our previous deep CNN model by optimizing the weights of the spectral samples in the training set. In “Classification method” section, we briefly describe the CNN model, while expounding the sample weight optimization strategy and the data partition scheme in detail.

### CNN model

**Fig. 3:**
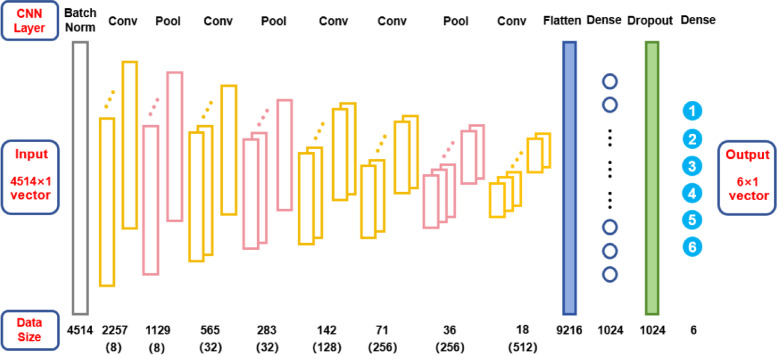
The architecture of the deep CNN model constructed in Keras framework. Besides the input layer and output layer, the CNN model comprises one batch normalization layer, five convolutional layers, three pooling layers, one flatten layer, two dense layers, and one dropout layer. At the bottom, there are two rows of values: each value in the upper row denotes the output data size of the corresponding layer; each value in the lower row (i.e. in the parentheses) denotes the number of the corresponding feature maps.

CNN is a cutting-edge deep learning method within the artificial neural network paradigm, with its core techniques being the convolution and pooling operations^28,29^. Implemented in Python, the deep CNN model is constructed using Keras Sequential API, with the model architecture displayed in Fig. 3.

In addition to the input layer and output layer, the deep CNN model consists of one batch normalization layer, five convolutional layers, three pooling layers, one flatten layer, two dense layers, and one dropout layer. The batch normalization layer can standardize the intensity distribution across the input spectral samples. Hence our data preprocessing pipeline omits standard normal variate transformation, as this step cannot effectively improve the performance of the CNN model (already incorporating batch normalization) in our practice. The convolutional layers are responsible for identifying and extracting the underlying features from the spectral data. The pooling layers reduce data dimensionality and introduce translation invariance, thereby enhancing the model robustness. The flatten layer converts the high-dimensional feature maps into a one-dimensional vector to facilitate its connection to the subsequent dense layers. The dense layers establish complex nonlinear mappings between the extracted features and the classification results. The dropout layer randomly deactivates a portion of the neurons at a specified rate during the training to prevent overfitting, and the dropout rate is set to 0.2 in this work.

The input and output dimensions of the CNN model are presented in Fig. 3. Each input data is a 4514 × 1 vector, corresponding to the size of each spectral sample. Each output data is a 6 × 1 vector[*C*_1_, *C*_2_, … *C*_6_]^T^, and *C*_*j*_ (*j* = 1, 2, … 6) denotes the probability that the input spectrum belongs to the *j*th class. For the model training, the Adam algorithm is used as the iteration optimizer, and the cross-entropy function is employed as the loss function for the classification task.

### Spectral sample weight optimization strategy

In a typical process of CNN model training, each spectral sample in the training set is assigned an equal weight by default. Of course, such an equal weight scheme is not wrong, but it has not fully considered the influence of spectral samples acquired at different distances on the model training. The two major effects are specifically analyzed as follows. Firstly, the SNR values of the spectral samples can influence the training effectiveness of the CNN model. The training set spectra detected at shorter distances have relatively higher SNR values and hence deserve more attention from the CNN model, while those spectra detected at longer distances have relatively lower SNR values and hence should receive less attention. Secondly, the relative distance between the training spectral samples and the testing spectral samples also plays a role in the training effectiveness. For a certain testing set and the corresponding detection distance, the training set spectra detected at distances closer to the testing set distance have higher training–testing data similarity and hence should be prioritized by the model, whereas those spectra detected at distances more distant from the testing set distance should be given less emphasis.

It is important to emphasize that enhancing SNR of the spectra is not the motivation of this study. Instead, through the optimization of the training sample weights, we aim to guide the CNN model to allocate its attention more reasonably during the training process, and thereby improve the model classification performance. Specifically, the weight of each training set spectral sample is elaborately designed according to both the absolute distance and the relative distance.

**Fig. 4:**
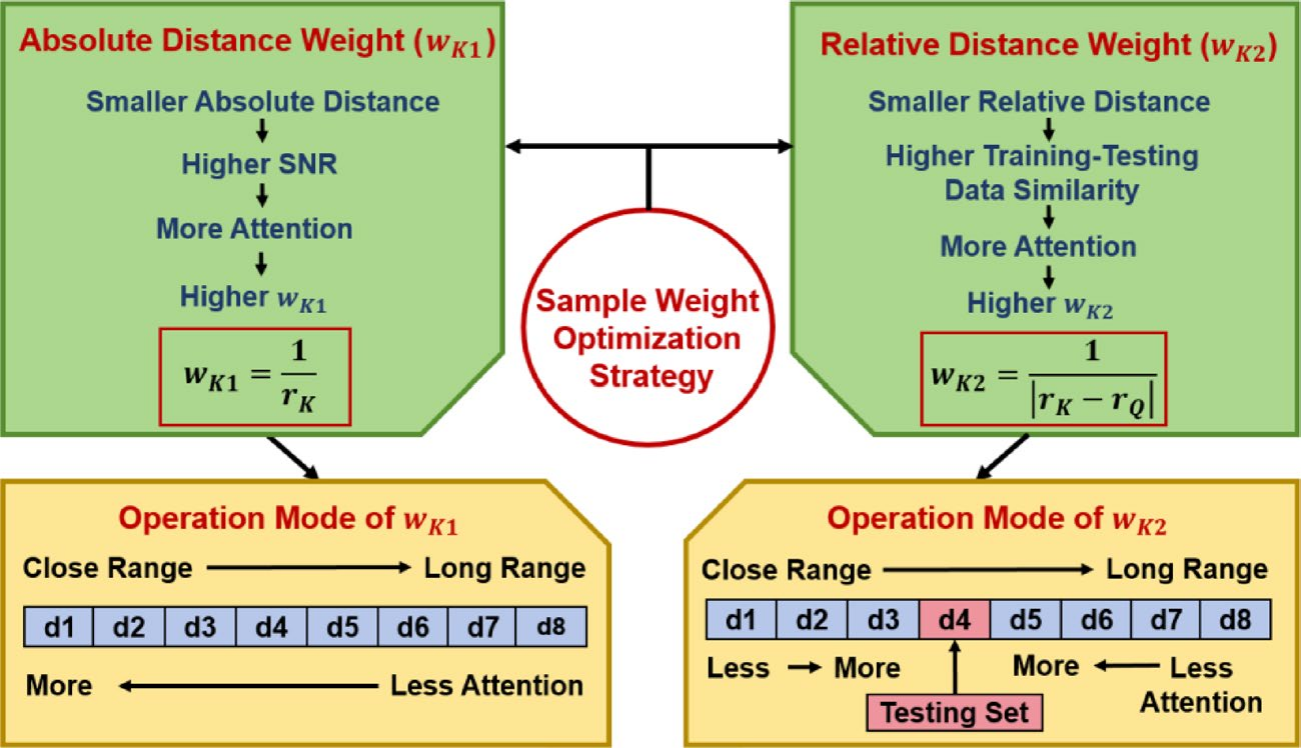
Diagram of the spectral sample weight optimization strategy. The upper part demonstrates the principle of weight optimization, with the left and right modules showing the calculation logic of the absolute weight and the relative weight, respectively. The lower part demonstrates the specific operation mode, with the left module illustrating the operation mode of the absolute weight, and the right module illustrating the operation mode of the relative weight (e.g., taking d4 as the testing set).

Figure 4 presents a flowchart summarizing the spectral sample weight optimization strategy proposed herein. Each training spectral sample weight consists of two parts: the absolute weight ($w_{K1}$) and the relative weight ($w_{K2}$). The method for calculating $w_{K1}$ and $w_{K2}$ are described by the following equations. For clarity, in Table 3 we list the involved symbols, along with their definitions and value ranges.

**Table 3:** The symbols used in Eqs. (1) through (4).

Symbol	Definition	Value range
*Q*	An integer between 1 and 8	1, 2, 3, 4, 5, 6, 7, 8
*K*	An integer between 1 and 8, excluding *Q*	1, 2, 3, 4, 5, 6, 7, 8 (*K* ≠ *Q*)
d*Q*	The testing set, one of the eight spectra datasets	d1, d2, d3, d4, d5, d6, d7, d8
d*K*	The training set, one of the seven spectra datasets other than d*Q*	d1, d2, d3, d4, d5, d6, d7, d8 (d*K* ≠ d*Q*)
*r*_*Q*_	The detection distance corresponding to d*Q*	2.0 m, 2.3 m, 2.5 m, 3.0 m, 3.5 m, 4.0 m, 4.5 m, 5.0 m
*r*_*K*_	The detection distance corresponding to d*K*	2.0 m, 2.3 m, 2.5 m, 3.0 m, 3.5 m, 4.0 m, 4.5 m, 5.0 m (*r*_K_ ≠ *r*_*Q*_)

When using d*Q* as the testing set, the $w_{K1}$ value of the spectra in d*K* is calculated by Eq. (1)

1 $$w_{K1} = \frac{1}{{r_{K} }},\;\;K = 1,2, \ldots Q - 1, Q + 1, \ldots 8$$

The $w_{K2}$ value of the spectra in d*K* is calculated by Eq. (2)

2 $$w_{K2} = \frac{1}{{\left| {r_{K} - r_{Q} } \right|}},\quad Q = 1,2, \ldots 8, \;\;{\text{and}}\;\;K = 1,2, \ldots Q - 1,Q + 1, \ldots 8$$

Considering the above two factors, the total weight $w_{K}$ of the spectra in d*K* can be written as Eq. (3)

3 $$\begin{aligned} w_{K} & = w_{K1} + w_{K2} = \left( {\frac{1}{{r_{K} }} + \frac{1}{{\left| {r_{K} - r_{Q} } \right|}}} \right), \\ & \quad Q = 1, 2, \ldots 8,\;\; {\text{and}}\;\;K = 1, 2, \ldots Q - 1, Q + 1, \ldots 8 \\ \end{aligned}$$

In order to make every sample weight a dimensionless value between 0 and 1, we add a final normalization step, with the normalized total weight $w_{K}^{\prime}$ defined in Eq. (4)

4 $$w_{K}^{\prime} = \frac{{w_{K} }}{{\mathop \sum \nolimits_{K} w_{K} }},\quad K = 1, 2, \ldots Q - 1,Q + 1, \ldots 8$$

For instance, we use d4 as the testing set and the remaining spectra datasets as the training set. The $w_{K}$ value of the spectra in d1 is calculated by $w_{1} = w_{11} + w_{12}$ = $\frac{1}{{r_{1} }} + \frac{1}{{\left| {r_{1} - r_{4} } \right|}} = \frac{1}{2} + \frac{1}{{\left| {2 - 3} \right|}}$ = 1.500 m^−1^. Similarly, the $w_{K}$ values for the other training spectra datasets can be obtained: 1.863 m^−1^ (d2), 2.400 m^−1^ (d3), 2.286 m^−1^ (d5), 1.250 m^−1^ (d6), 0.889 m^−1^ (d7), and 0.700 m^−1^ (d8). Then the $w_{K}^{\prime}$ value of the spectra in d1 can be calculated by $w_{1}^{\prime}$ = $\frac{{w_{1} }}{{\mathop \sum \nolimits_{K = 1}^{3} w_{K} + \mathop \sum \nolimits_{K = 5}^{8} w_{K} }}$ = $\frac{1.500}{{1.500 + 1.863 + 2.400 + 2.286 + 1.250 + 0.889 + 0.700}}$ = 0.138. The $w_{K}^{\prime}$ values for the other datasets can be acquired likewise: 0.171 (d2), 0.220 (d3), 0.210 (d5), 0.115 (d6), 0.082 (d7), and 0.064 (d8). In Table 4, the calculated $w_{K}^{\prime}$ values for eight different testing set conditions are collectively displayed.

**Table 4:** The spectral sample weight values for different testing set conditions.

Testing set ↓	Training set →							
	d1	d2	d3	d4	d5	d6	d7	d8
d1	×	0.364	0.232	0.129	0.092	0.072	0.060	0.051
d2	0.270	×	0.380	0.124	0.079	0.059	0.048	0.040
d3	0.181	0.394	×	0.169	0.093	0.066	0.052	0.044
d4	0.138	0.171	0.220	×	0.210	0.115	0.082	0.064
d5	0.111	0.121	0.133	0.222	×	0.214	0.116	0.082
d6	0.099	0.101	0.105	0.132	0.226	×	0.219	0.118
d7	0.095	0.094	0.095	0.106	0.136	0.239	×	0.233
d8	0.108	0.105	0.104	0.108	0.124	0.162	0.289	×

As stated above, each training set spectral sample is assigned a corresponding weight value. During the training process, both the sample data and their associated weight values are fed into the CNN model. In the Keras framework, the weight values are input into the model via a parameter called “sample_weight”.

As a matter of fact, the iteration during the training is carried out along the direction of minimizing the loss function. For the underlying operations in Python, assigning weights to the individual spectral samples is essentially weighting their contribution to the loss function. To be more specific, the classification error (loss) yielded from each sample would be multiplied by its corresponding weight value. Consequently, misclassifying a high-weight sample results in a more substantial increase in the loss. This mechanism guides the CNN model to prioritize learning from those “important” samples during the training.

### Dataset partition schemes

This work adopts a training–validation–testing scheme to divide the whole dataset. In this scheme, there exists an independent validation set so that efficient hyperparameter tuning can be implemented, and the testing set remains a thoroughly independent set for final evaluation. Considering there are eight distances and 37 target samples, we divide the training set, validation set, and testing set with a ratio of 6:1:1 in the distance dimension, and 25:6:6 in the target sample dimension.

In the present study, we have employed three partition schemes, namely Scheme1, Scheme2, and Scheme3. Regarding the distance dimension, d3 is used as the validation set in all three partition schemes, while the testing set varies with the scheme: d2 is used as the testing set for Scheme1, d4 for Scheme2, and d7 for Scheme3. According to the description in “LIBS datasets” section, one can find that such a selection strategy ensures that the datasets in every distance range (i.e. short-range, medium-range, and long-range) have the chance to serve as the testing set. The training set naturally comprises the six datasets other than the validation set and the testing set.

As for the target sample dimension, the partition strategy remains consistent across all three schemes. The validation set includes six target samples, namely Sample No. 2, 3, 9, 20, 24, and 31, with one sample selected from each of the six classes. Another six target samples, namely Sample No. 13, 19, 25, 26, 29, and 37, form the testing set, which also contains one representative sample from each class. The rest 25 target samples constitute the training set. Such a design ensures both the validation set and the testing set provide balanced representation across all six classes.

**Fig. 5:**
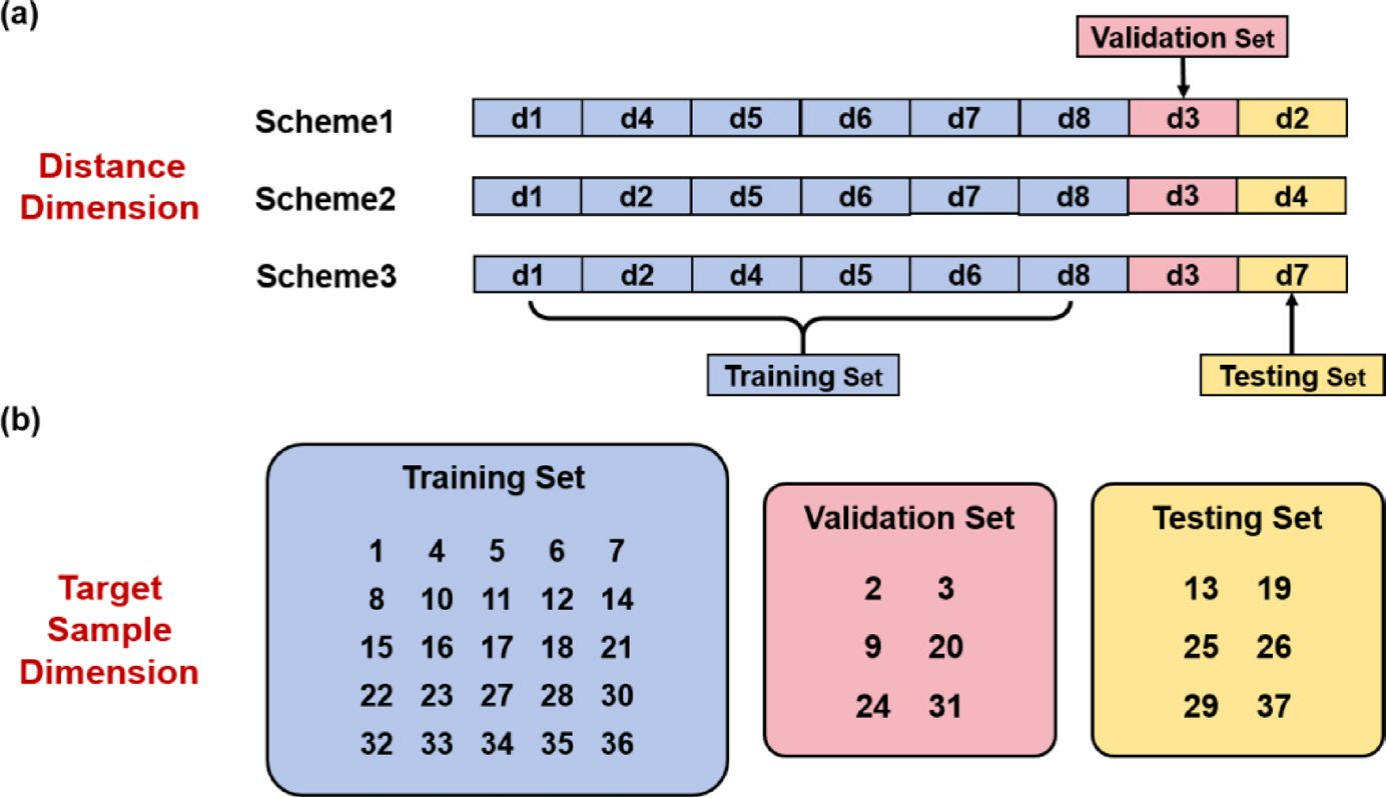
The dataset partition schemes for the model training, validation and testing. The training set (purple background), validation set (pink background), and testing set (yellow background) are divided with a ratio of 6:1:1 in the distance dimension, and 25:6:6 in the target sample dimension. (**a**) The partition mode in the distance dimension. (**b**) The partition mode in the target sample dimension.

The overall dataset partition situation is illustrated in Fig. 5. Notably, there is no intersection between the training, validation and testing sets in either the distance dimension or the target sample dimension. Therefore, this classification task is challenging enough to effectively evaluate the generalization ability of the deep CNN model.

## Results and discussion

In “Results and discussion” section, the approach to tuning the CNN hyperparameters, i.e. Bayesian optimization, is briefly described. Subsequently, the model performance metrics before and after applying the spectral sample weight optimization are compared. In addition, the results of ablation study are presented to further reveal the individual roles of absolute weight and relative weight. Finally, some important points and future prospects are discussed.

All computations were performed using the following hardware and software configuration: CPU: 16-core AMD EPYC 9354; GPU: NVIDIA GeForce RTX 4090; Python 3.10.12; Keras 3.0.

Prior to presenting the results, we designate the following four sample weighting schemes for clarity. The “Equal Weight” scheme refers to the default weighting strategy used in our previous work; the “Total Weight” scheme denotes the sample weight optimization strategy expounded in “Spectral sample weight optimization strategy” section; the “Absolute Weight” scheme indicates the exclusive use of the absolute weight part; and the “Relative Weight” scheme represents the exclusive use of the relative weight part. While the “Equal Weight” and the “Total Weight” schemes are adopted throughout in “Results” section, the “Absolute Weight” and the “Relative Weight” schemes are only utilized in the ablation study (In “Ablation study of weight contributions” section).

### Results

#### CNN hyperparameter tuning

In this work, three key hyperparameters for the CNN model training have been carefully tuned, i.e. the initial learning rate, the number of epochs, and the batch size.

Using the training set and validation set in each of the three dataset partition Schemes, we have applied the Bayesian optimization algorithm^30^ to search the optimal hyperparameters. For the Bayesian optimization, 50 searching iterations are performed in both the Equal Weight scheme and the Total Weight scheme, with the mean square error (MSE) employed as the optimization indicator.

The hyperparameter tuning ranges and the optimized hyperparameters acquired from the Bayesian optimization are listed in Table 5. It is worth noting that the number of training epochs in the Equal Weight scheme is different from that in the Total Weight scheme (especially for the dataset partition Scheme3). This difference leads to the disparity in the training time of the two weighting schemes, as will be stated in “Classification performance comparison” section.

**Table 5:** The hyperparameter tuning ranges and optimized hyperparameters for the three dataset partition Schemes and two sample weighting schemes.

Hyperparameters			Learning rate	Epoch number	Batch size
Hyperparameter tuning range			[1E−5, 1E−3]	[256, 1024]	[500, 1000]
Optimized value	Partition Scheme1	Equal weight	1.00E−3	343	967
		Total weight	2.86E−4	344	997
	Partition Scheme2	Equal weight	9.28E−4	271	502
		Total weight	1.00E−3	266	557
	Partition Scheme3	Equal weight	6.88E−4	284	517
		Total weight	6.20E−4	398	998

#### Classification performance comparison

In this study, the primary metric to evaluate the model classification performance is the accuracy on the testing set. Accuracy denotes the ratio between *N*_*corr*_ and *N*_*tot*_, where *N*_*tot*_ means the total number of spectral samples in the testing set (*N*_*tot*_ = 360) and *N*_*corr*_ indicates the number of correctly classified spectral samples. Since the number of target samples in each class is not ideally balanced (varying from three to eight), we have additionally utilized three supplementary metrics, namely precision, recall, and F1-score (all are macro-averaged), to provide a more comprehensive evaluation of model performance on the imbalanced dataset^31^.

**Fig. 6:**
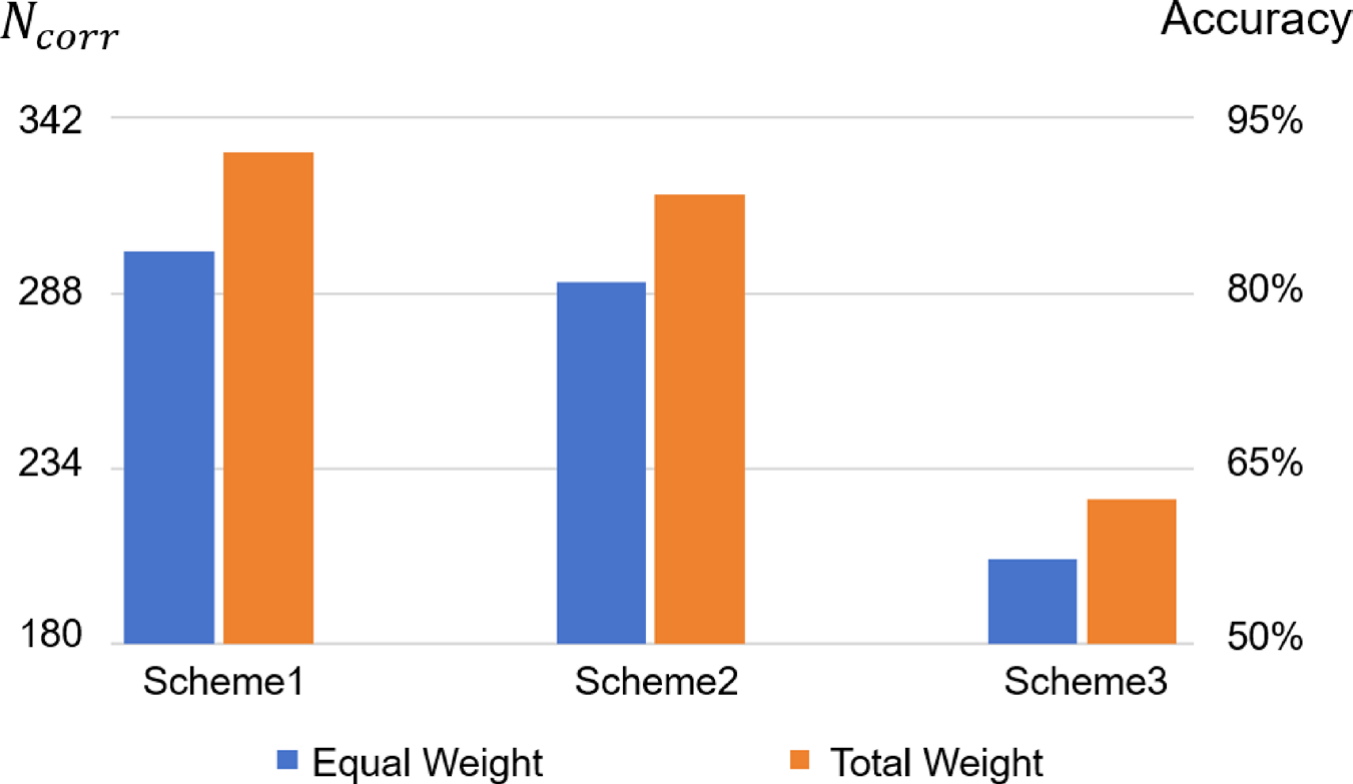
Comparison of $N_{corr}$ (left Y-axis) and accuracy (right Y-axis) by the deep CNN model across three dataset partition Schemes: equal weight scheme (blue bars) versus total weight scheme (orange bars).

On the three dataset partition Schemes, we compare the model testing performance metrics before and after applying the sample weight optimization strategy. Figure 6 shows the accuracy comparison of the two weighting schemes across the three dataset partition Schemes, with both *N*_*corr*_ and accuracy values exhibited. It can be found that the model adopting the sample weight optimization strategy has stronger performance across all the three Schemes. To be more specific, the proposed Total Weight scheme achieves accuracy values of 92.06%, 88.39%, and 62.39% across the three Schemes, representing improvements of 8.45, 7.39, and 5.11 percentage points over the default Equal Weight scheme, respectively.

In Table 6, the comparison results of all the four evaluation metrics are listed (note that each displayed value represents the mean of the results from five independent runs).

**Table 6:** The testing performance comparison between the equal weight scheme and the total weight scheme.

Partition scheme	Weighting scheme	Accuracy (%)	Precision	Recall	F1-score
Scheme1	Equal weight	83.61	0.845	0.836	0.813
	Total weight	92.06	0.932	0.921	0.916
Scheme2	Equal weight	81.00	0.844	0.810	0.796
	Total weight	88.39	0.905	0.884	0.888
Scheme3	Equal weight	57.28	0.648	0.573	0.536
	Total weight	62.39	0.692	0.624	0.587

The precision, recall, and F1-score metrics acquired from the Total Weight scheme consistently outperform the Equal Weight scheme on all three data partition Schemes. The mean improvements (averaging over the three Schemes) in precision, recall, and F1-score reach 6.4, 7.0 and 8.2 percentage points, respectively. These comprehensive results demonstrate that the spectral sample weight optimization strategy can effectively enhance the classification performance of the deep CNN model.

Beyond the testing accuracy aspect, the time cost of training is also an important aspect for a comprehensive model assessment. Table 7 presents a comparison of the number of training epochs, total training time, and time per epoch. The total training time is primarily influenced by the number of epochs. For example, in partition Scheme3, the total time of the Total Weight scheme apparently exceeds that of the Equal Weight scheme. However, the computational costs of the two weighting schemes are nearly identical regarding the time per epoch. Even for Scheme3, the time per epoch of the Total Weight scheme is merely about 0.1 s longer. The results indicate that the proposed weight optimization strategy introduces negligible extra computational cost to the training process.

**Table 7:** Comparison of the time cost across the three dataset partition schemes: equal weight scheme versus total weight scheme.

Partition scheme	Weighting scheme	Epoch number	Total time (s)	Time per epoch (s)
Scheme1	Equal weight	343	85	0.248
	Total weight	344	86	0.250
Scheme2	Equal weight	271	42	0.155
	Total weight	266	41	0.154
Scheme3	Equal weight	284	43	0.151
	Total weight	398	98	0.246

#### Ablation study of weight contributions

In order to investigate the individual contributions of the absolute weight part and the relative weight part in the proposed weight optimization strategy, we have conducted an ablation study based on the three dataset partition Schemes.

**Fig. 7:**
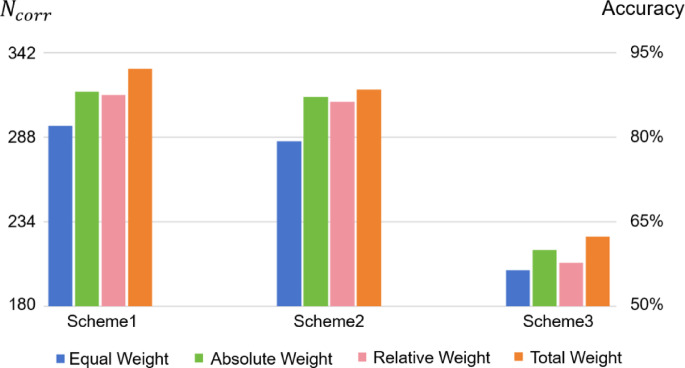
Comparison of $N_{corr}$ (left Y-axis) and accuracy (right Y-axis) by the deep CNN model across three dataset partition Schemes: equal weight scheme (blue bars) versus absolute weight scheme (green bars) versus relative weight scheme (pink bars) versus proposed total weight scheme (orange bars).

In the ablation study, the model hyperparameters for the four weighting schemes (Equal Weight, Absolute Weight, Relative Weight, and Total Weight) within each dataset partition Scheme are set to be identical for fair comparison. Specifically, in each partition Scheme, all the four weighting schemes adopt those hyperparameters of the Total Weight scheme obtained via the Bayesian optimization. Similar to Fig. 6, we illustrate the accuracy comparison of the four weighting schemes in Fig. 7, with both *N*_*corr*_ and accuracy values presented.

The comparison results of all the four evaluation metrics in the ablation study are exhibited in Table 8.

**Table 8:** Ablation study results on the three dataset partition schemes.

Partition scheme	Weighting scheme	Accuracy (%)	Precision	Recall	F1-score
Scheme1	Equal weight	81.94	0.796	0.819	0.788
	Absolute weight	88.00	0.894	0.876	0.869
	Relative weight	87.50	0.891	0.875	0.868
	Total weight	92.06	0.932	0.921	0.916
Scheme2	Equal weight	79.28	0.809	0.793	0.766
	Absolute weight	87.11	0.888	0.871	0.866
	Relative weight	86.22	0.878	0.862	0.857
	Total weight	88.39	0.905	0.884	0.888
Scheme3	Equal weight	56.44	0.630	0.564	0.532
	Absolute weight	60.00	0.655	0.600	0.560
	Relative weight	57.72	0.637	0.577	0.537
	Total weight	62.39	0.692	0.624	0.587

Across all the three dataset partition Schemes, either the Absolute Weight scheme or the Relative Weight scheme surpasses the Equal Weight scheme regarding the four evaluation metrics, indicating that both the absolute weight part and the relative weight part can make positive contribution to the weight optimization strategy. This conclusion can also be verified by the fact that the Total Weight scheme achieves the best performance among the four weighting schemes.

### Discussion

#### Further analysis on classification performance comparison

As stated in “Classification performance comparison” section, on the three dataset partition Schemes, the Total Weight scheme obtains accuracy improvements of 8.45, 7.39, and 5.11 percentage points over the Equal Weight scheme, respectively. Recalling the definitions of Scheme1, Scheme2, and Scheme3, it can be inferred that the performance gain from our sample weighting strategy is more pronounced on higher SNR testing sets. For example, we can achieve the most significant gain on Scheme1, as its testing set (d2) has the highest SNR spectra among the three testing sets (d2, d4, and d7). For Scheme3, it suffers from double penalty: the baseline performance is already low, and and it also receives the smallest marginal improvement from the proposed weighting strategy.

This phenomenon is a consequence of our compound weighting strategy, which considers both data quality (absolute weight) and domain relevance (relative weight). The strategy shapes a model that excels at analyzing high-quality (high-SNR) data, and the data most similar to the testing data.

On a high-SNR testing set (short-distance data), the CNN model enjoys a synergistic advantage. Both the absolute weight part and the relative weight part guide the model to specialize in processing high-SNR data. Hence the high-SNR testing data allow the model’s specialized capability to flourish, resulting in a maximal performance gain.

On a low-SNR testing set (long-distance data), however, the CNN model actually faces a dilemma. The relative weight part directs the model to focus on the relevant long-distance data, but the inherently low SNR of these samples limits the useful information available for learning and prediction. Meanwhile, the absolute weight part still aims to direct the model to focus on the high-SNR data, leading to a contradiction in the learning strategy. Since the impact of the absolute weight is not equal to that of the relative weight (the Absolute Weight scheme always achieves better performance than the Relative Weight scheme, as demonstrated in Table 8), the net effect of the weighting scheme will not be zero. Thus, the model can still achieve a performance gain, but the gain is limited.

#### Future prospects

Despite the performance improvement achieved by the proposed spectral sample weighting strategy, there are some limitations in this work. In future research, the following points can be considered for further enhancement.

1. The LIBS spectra in this study were all collected in laboratory environments. However, unlike the clean target samples in the laboratory, the target samples in the Martian atmosphere can be covered by dust on their surfaces. As the detection distance increases, the laser intensity reaching the target sample may weaken, resulting in reduced ablation efficiency. This may require more laser pulses to ablate the dust on the sample surfaces. Taking the data collection mode in this study as an example (i.e. 60 spectra per target sample), one might find that for close detection 10 pulses are enough to remove the surface dust and reach the actual target sample, whereas for remote detection 30 or even more pulses are necessary. Therefore, if we consider the dust effect in Mars in-situ detection, those spectra collected at longer distances deserve further smaller weights in the model training. Moreover, the concept of “unequal weighting” could be generalized to the spectra collected at the same distance. For instance, among the 60 spectra collected at a certain distance, the initial spectra (e.g. the first 10 spectra) hitting the surface dust could be assigned smaller weights (even zero), while the latter spectra (e.g. the last 30 spectra) hitting the genuine target sample could receive greater weights.
  Currently, we are able to collect LIBS spectra from dust-covered target samples using simulated Martian soil (e.g. JMSS-1 type), and such measurements can be conducted in a simulated Martian atmospheric environment (95.73% CO_2_, 2.67% N_2_, and 1.6% Ar, 876 Pa, − 16 °C) based on our MarSDEEP facility^32^. However, the current number of spectra acquired in the simulated Martian environment and on the dust-covered target samples are not sufficient to train an effective deep learning CNN model. In future work, more such spectra will be acquired and further data analysis will be carried out, thereby providing support for the interpretation of authentic in-situ LIBS spectra detected by MarSCoDe on Mars.
2. This study adopts a sample weighting strategy that combines the absolute weight and the relative weight. However, the current combination strategy simply adds the two parts of weights, lacking trials in other possible combination strategies. In future work, more combination strategies can be explored to potentially achieve even better model performance. Moreover, while the proposed weighting strategy has been empirically validated on a conventional CNN model, its applicability to other advanced deep learning architectures, such as ResNet^33^ and Transformer^34^, will be an important direction of subsequent research.
3. The fusion of multiple dimension information is a developing trend for LIBS chemometrics model design. Leveraging information in addition to the spectra themselves may maximize the utilization efficiency of every single spectrum, thereby compensating for the scarcity of in-situ spectra to some extent. In future work, one can consider incorporating additional information (image, characteristic parameters, etc.) of the laser ablation/plasma evolution processes into the spectral information. The additional information can be acquired from experiments^35^ and/or numerical simulations^36,37^. By utilizing the multi-dimensional information, one may further upgrade the spectral sample weight optimization strategy.
4. Since the laboratory LIBS spectra and Mars in-situ LIBS spectra can have considerable profile discrepancies, we have proposed a novel LIBS chemometrics method combining deep learning (CNN) and transfer learning in one of our recent studies^38^. The transfer learning is in pretrained-model-based pattern, and the performance of the final CNN model in analyzing Mars in-situ spectra is highly dependent on the performance of the pretrained model (trained by the laboratory spectra). The spectral sample weight optimization strategy may enhance the performance of the pretrained CNN model, thereby improving the competence of the final model after transfer learning.

The above points will be considered in our future work and are anticipated to further promote the accuracy of Mars in-situ LIBS data analysis.

## Conclusion

This study has proposed a novel spectral sample weight optimization strategy to enhance the classification performance of a deep CNN for LIBS multi-distance mixed spectra. Unlike the default equal weight scheme, the new method assigns a tailored weight to each training set sample based on both its absolute detection distance and its relative distance to the testing set.

Experimental results on an eight-distance LIBS dataset demonstrate that the weight optimization scheme can well increase the model classification accuracy, achieving a maximum improvement of 8.45 percentage points compared to the equal weight scheme. Beyond accuracy, three supplementary metrics also show the superiority of the proposed strategy: the precision, recall and F1-score can be averagely increased by 6.4, 7.0 and 8.2 percentage points, respectively. The ablation study confirms the positive contributions of both the absolute weight part and the relative weight part, with the former playing a more influential role. Moreover, observations of the training time per epoch indicate that the weight optimization strategy hardly introduces extra computational overhead to the training process.

More work will be carried out to further enhance the applicability of this new scheme to analyzing Mars in-situ LIBS spectra, such as collecting more spectra in simulated Martian environments and on dust-covered samples, exploring more different weighting strategies and advanced deep learning models, utilizing multi-dimensional data fusion, integrating deep learning and transfer learning, etc. In addition to the planetary exploration, the proposed sample weighting methodology is also promising for other LIBS field detection scenarios where the detection distance variation is required.

## Data Availability

The datasets generated during and/or analysed during the current study are not publicly available due to data confidentiality but are available from the corresponding author on reasonable request.
